# Are congenital heart defects connected to more severe attention-deficit/hyperactivity disorder?: A systematic review and meta-analysis

**DOI:** 10.1097/MD.0000000000036193

**Published:** 2023-11-24

**Authors:** Mohammed Tarek Hasan, Mahmoud Shaban Abdelgalil, Merihan A. Elbadawy, Amr Mahmoud Elrosasy, Ali Elkhadragy, Mahmoud El Garhy, Ahmed K. Awad

**Affiliations:** a Faculty of Medicine, Al-Azhar University, Cairo, Egypt; b Faculty of Medicine, Ain Shams University, Cairo, Egypt; c Helwan University Hospital, Cairo, Egypt; d Alexandria University, Faculty of Medicine, Cairo, Egypt.

**Keywords:** attention-deficit/hyperactivity disorder, congenital heart defects, meta-analysis

## Abstract

**Background:**

Congenital heart defects (CHDs) are the most common cause of birth defect-related infant morbidity and mortality, affecting 1% of 40,000 births per year in the United States. On the other side, the etiology of attention-deficit/hyperactivity disorder (ADHD) is multifactorial. Multiple studies have found that cardiac surgery patients have higher morbidity of having this disorder. Many studies have investigated the prevalence of ADHD in different subtypes of CHD, but few have focused on the severity of ADHD symptoms. Thus, we conducted this systematic review and meta-analysis to investigate the severity of ADHD symptoms in CHD patients.

**Methods:**

We searched PubMed, Embase, Scopus, and Web of Science were searched from inception to March 6, 2023 without any restrictions. We included observational studies published in English language that evaluated burden of symptom of ADHD in CHD patients. Moreover, the standardized mean difference (SMD) for continuous outcomes with 95% confidence interval (CI) was pooled. *P*-values <.05 are considered as significant, and we performed all statistical analyses using RevMan software Version 5.4.1.

**Results:**

Eight studies were included in our review with a total number of 120,158 patients. CHD was associated with a statistically significant increase in both ADHD index T score and ADHD Hyperactivity-Impulsivity Subscale (informant) with (SMD = 0.65, 95% CI [0.40, 0.90], *P* < .00001, I^2^ = 81%) and (SMD = 0.16, 95% CI [0.04, 0.28], *P* = .008, I^2^ = 0%). Regarding ADHD Inattention Subscale (informant), the pooled data showed that a significant increase of this score in the CHD group (SMD = 0.25, 95% CI [0.13, 0.37], *P* < .001, *I*^2^ = 0%), and ADHD Combined Score (informant) showed a significant increase of this score in the CHD group (SMD = 0.23, 95% CI [0.11, 0.35], *P* = .0002, I^2^ = 0%).

**Conclusion:**

Our study revealed a strong association between CHD and not only ADHD, but also the severity of ADHD, making early diagnosis of ADHD in children with CHD a mandatory step in the clinical evaluation practice to improve these children on both clinical and psychological aspects.

## 1. Introduction

Congenital heart defects (CHDs) are the most common cause of birth defect-related infant morbidity and mortality, affecting 1% of 40,000 births per year in the United States. CHDs related death often occurs in babies <28 days old (neonatal period)). CHDs cause about 4.2% of all neonatal deaths. Between 1999 to 2006, the United States recorded about 41,494 deaths related to CHDs, which means that CHDs can either be the cause of the death or contribute to death somehow.^[1]^

Recent improvements in medical treatments, surgical procedures, imaging, and pediatric intensive care helped more than 90% of congenital heart disease (CHD) children reach adulthood and decreased the total mortality rate by 31%in the previous 20 years.^[2,3]^ This changes our focus on CHD from mortality to morbidity, where although babies born with CHD now have a longer life expectancy and better quality of life, they are more likely to have psychological distress, neurocognitive deficits, and social challenge. Studies reported that approximately 30% had mood or anxiety disorders and 20% showed symptoms comparable with posttraumatic stress disorder.

Children and adolescents with CHD are at an increased risk of neurodevelopmental abnormalities, increasing in frequency and severity as the disease complexity increases. There is an increase in the prevalence of neurodevelopmental disabilities, deficits in behavior, language, and speech disorders, attention-deficit/hyperactivity disorder (ADHD), and increased use of services among young patients with CHD.^[4]^

Even though the etiology of ADHD is multifactorial, multiple studies found that cardiac surgery patients have higher morbidity of having this disorder, particularly after aortic arch repairs and corrections of great artery transposition, thus explains why CHDS children have a 30% greater chance of inattention and hyperactivity disorder than healthy persons. Approximately half of the surgically treated children require remedial school services by reaching adolescence, ADHD symptoms in cardiac patients are frequently misdiagnosed and under-treated.^[5,6]^

Several studies have tried to understand the relationship between ADHD and CHDs, where some studies suggest that complex CHD children have abnormal cerebral blood flow, which was already altered during pregnancy and peripartum periods, leading to a delay in brain maturation. Additional postponed correction of cardiac defects increases the risk for ADHD development due to hypoxemia and can further damage the highly oxygen-sensitive regions of the prefrontal cortex.^[7]^ Other risk factors include lower birth weight, longer duration of deep hypothermic circulatory arrest, and repeated surgeries as ADHD symptoms are worsened by repeated exposure to surgery, anesthesia, and inflammatory responses.^[8,9]^

To date, many studies investigated the prevalence of ADHD in different subtypes of CHD but little focused on the severity of ADHD symptoms. Also, the studies are either based on small groups of children or are limited to a single heart lesion. We conducted this systematic review and meta-analysis to investigate the severity of ADHD symptoms in CHD patients.

## 2. Method

We conducted this systematic review and meta-analysis in accordance with the Meta-Analysis of Observational Studies in Epidemiology^[10]^ and Preferred Reporting Items for Systematic Reviews and Meta-Analyses^[11]^ guidelines. The study protocol was registered on open science framework (Registration DOI: 10.17605/OSF.IO/ZH2YX).

### 2.1. Search strategy and study selection

PubMed, Embase, Scopus, and Web of Science were searched from inception to March 6, 2023 without any restrictions. We generated our search strategy using Mesh database, and the search strategy is formed of combinations of the following search terms (“attention deficit disorder with Hyperactivity” OR ADHD OR “Hyperkinetic Syndrome” OR ADDH OR “Attention Deficit Hyperactivity Disorder” OR “Attention Deficit Disorder” OR “Minimal Brain Dysfunction”) AND (“Heart Abnormality” OR “Congenital Heart Defect” OR “Malformation Of Heart” OR “Congenital Heart Disease”). We included observational studies published in English language that evaluated burden of symptom of ADHD in CHD patients. No restrictions regarding date of publication. We excluded studies with insufficient data for extraction, reviews, book chapters, thesis, editorial, letters, conference papers, and non-English studies, literature reviews, and meta-analysis. Two independent authors screened the articles in 3 stages; title, abstract, and full text on an excel sheet. Another independent author resolved any disagreements.

### 2.2. Risk of bias assessment

We used the Newcastle-Ottawa scale^[12]^ to assess the risk of bias within the included observational studies. This scale is evaluating 3 essential domains:

(a) Selection of the study subjects.
(b) Comparability of groups on baseline characteristics and other important confounders.
(c) exposure or outcome and follow up.

Each article was evaluated by 2 independent authors and any disagreements were resolved by another author. The number of included studies were <10, so we could not assess publication bias using Egger test for funnel plot asymmetry.

### 2.3. Data extraction

Two independent authors extracted the data in the form of the following domains: (1) *summary and baseline characteristics including*: design, number of participants, gender, age, birth weight, gestation, family social status, and IQ. (2) *Study outcomes*: The authors extracted the following outcomes: ADHD index T score, ADHD Inattention Subscale, ADHD Hyperactivity-Impulsivity Subscale, and ADHD Combined Score.

### 2.4. Data synthesis

The standardized mean difference (SMD) for continuous outcomes with 95% confidence interval (CI) was pooled. *P*-values <.05 are considered as significant. The I-square and *P*-value were used to determine heterogeneity. If the *P*-value was <.05 or the I-square was <50%, the analysis was judged heterogeneous. If heterogeneity was identified, a random-effect model was used, and a leave one out test was used to establish which study was producing the heterogeneity. We performed all statistical analyses using RevMan software Version 5.4.1.^[13]^

## 3. Results

### 3.1. Search results and baseline characteristics

**Figure 1.**
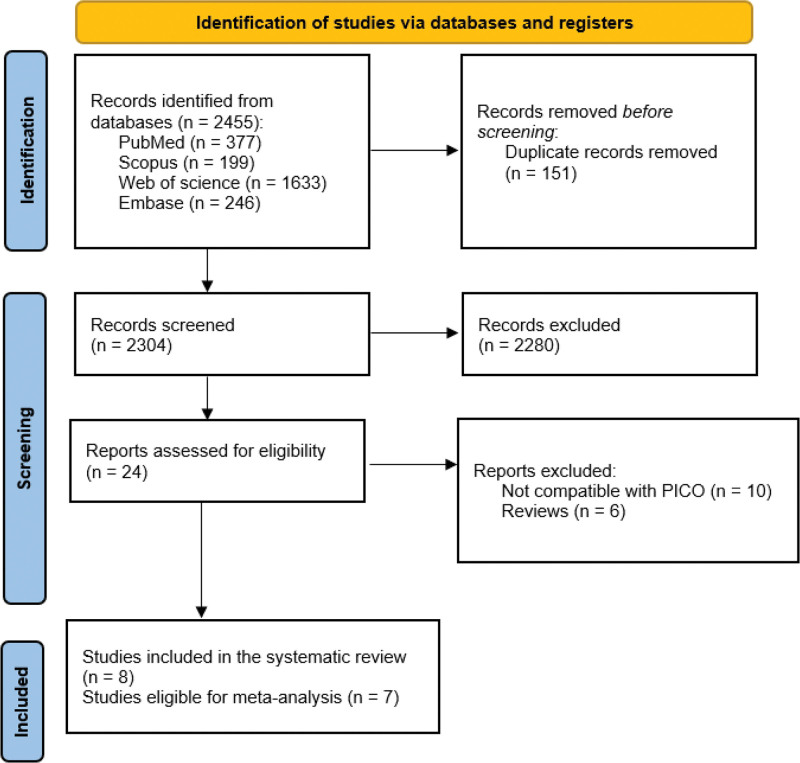
PRISMA flow diagram for included studies. PRISMA = Preferred Reporting Items for Systematic Reviews and Meta-Analyses.

Our database search on PubMed, Scopus, Web of Science, and Embase yielded 2455 studies. We removed 151 duplicate studies. We screened 2304 studies in 3 stages: title, abstract, and full text screening. After full text screening, we excluded 2280, and after full text screening, we excluded 16. Finally, we included 8 studies in our review; only 7 studies were eligible for the meta-analysis (Fig. 1).

The total sample size included in the meta-analysis was 120,158 (1869 with congenital heart diseases and 118,289 heathy control). We included children and young adults with mean age ranged from 4 to 26 years old, average IQ, and normal birth weight. The detailed summary of included studies and baseline characteristics reported in Table 1.

**Table 1:** Summary and baseline characteristics of included studies.

Study ID	Design	Groups	No	Male n(%)	Female n(%)	Age, y mean(SD)	Birth weight (kg) mean(SD)	Gestation (weeks) mean(SD)	Family social status mean(SD)	Full-scale IQ, mean(SD)
Czobor 2021	Cohort	CHD	80	40 (50)	40 (50)	12.41 (3.5)	_	_	_	_
		Control	53	24 (45.28)	29 (54.71)	11.79 (3.56)	_	_	_	_
DeMaso 2014	Cohort	CHD	139	104 (75)	35 (25)	16.1 (0.5)	_	_	_	98 (15)
		Control	61	30 (49)	31 (51)	15.3 (1.1)	_	_	_	108 (11)
DeMaso 2016	Cohort	CHD	156	95 (61)	61 (39)	14.5 (3.0)	3.3 (0.6)	38.9 (2.2)	50 (13)	91.6 (16.8)
		Control	111	59 (53)	52 (47)	15.3 (1.8)	3.5 (0.6)	39.6 (1.3)	53 (10)	108.3 (11.4)
Gonzalez 2020	Cohort	CHD	1164	661 (56.8)	503 (43.2)	4–9, 710 (61.0) 10–13, 244 (21.0) 14–17, 210 (18.0)	_	_	_	_
		Control	117,621	61,063 (52)	56,585(48)	4–9, 63 015 (53.6) 10–13, 29 461 (25.1) 14–17, 25 145 (21.4)	_	_	_	_
Holland 2017	Cohort	CHD	91	50 (55)	41 (45)	14.62 (1.18)	3.12 (0.68)	39.17 (2.34)	49 (11.9)	88.96 (22.5)
		Control	87	_	_	_	_	_	_	_
Holst 2019	Cohort	CHD	159	88 (55.3)	71 (44.7)	13 (1.8)	3.2 (0.7)	39.1 (2.6)	_	_
		Control	317	174 (54.9)	143 (45.11)	12.9 (1.7)	_	_	_	_
Lau-Jensen 2021	Case-control	CHD	80	23 (28.8)	57 (71.3)	26.6 (6)	_	_	_	_
		Control	39	14 (35.9)	25 (64.1)	25.3 (4.53)	_	_	_	_
Yamada 2013	Cohort	CHD	56	_	_	_	3.19	39	_	_
		Control	60	_	_	_	3.6	40	_	_

### 3.2. Quality assessments

All the included studies reported adequately the domain of selection of study subjects. All the included studies matched their participants according to baseline in comparability domain except Gonzalez 2020 and Holland 2017. Only 3 studies (Czobor 2021, DeMaso 2014, and DeMaso 2016) adequately reported the third domain. The detailed quality assessment reported in Table 2.

**Table 2:** ROB of included studies.

Study ID	1	2	3	4	5	6	7	8	Quality
Czobor 2021	*	*	*	*	**	*	*	*	High
DeMaso 2014	*	*	*	*	**	*	*	*	High
DeMaso 2016	*	*	*	*	*	*	*	*	High
Gonzalez 2020	*	*	*	*					Moderate
Holland 2017	*	*	*	*		*		*	Moderate
Holst 2019	*	*	*	*	*	*			Moderate
Lau Jensen 2021	*	*	*	*	*	*		*	High
Yamada 2013	*	*	*	*	**		*	*	High

### 3.3. ADHD index T score

ADHD index T score determine whether the patient have ADHD and rating the severity of the disease. Patients’ scores <60 are disease free but scores higher than 60 indicate ADHD, considering that the higher the score, the higher the disease severity. It consists of questions related to ADHD frequency of symptoms; the questions are based on the definition of ADHD provided by the Diagnostic and Statistical Manual of Mental Disorders (DSM-5; American Psychiatric Association, 2017).^[14]^

**Figure 2.**
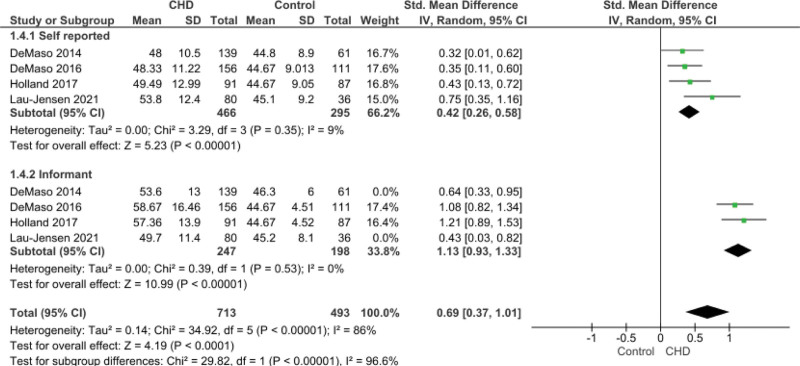
Forest plot illustrating pooled ADHD index T score. ADHD = attention-deficit/hyperactivity disorder.

This score reported in 4 studies. The pooled data showed a significant increase of the score in CHD group (SMD = 0.65, 95% CI [0.40, 0.90], *P* < .00001) but with high heterogeneity (*P* < .00001, I^2^ = 81%). Therefore, a subgroup analysis was performed according to the type of reporting to self-reported and informant. The pooled data of self-reported was homogenous and showed a significant increase in score in CHD group (SMD = 0.42, 95% CI [0.26, 0.58], *P* < .00001 (*P* = .35, I^2^ = 9%). The data of informant showed a significant increase of the score in CHD group too (SMD = 0.86, 95% CI [0.52, 1.19], *P* < .00001) but with high heterogeneity (*P* = .003, I^2^ = 78%); after removing DeMaso 2014 and Lau-Jensen 2021, The pooled data showed that significant increase of this score in CHD group (SMD = 1.13, 95% CI [0.93, 1.33], *P* < .00001). Pooled data were homogeneous (*P* = .53, I^2^ = 0%) (Fig. 2).

### 3.4. ADHD Hyperactivity-Impulsivity Subscale (informant)

**Figure 3.**
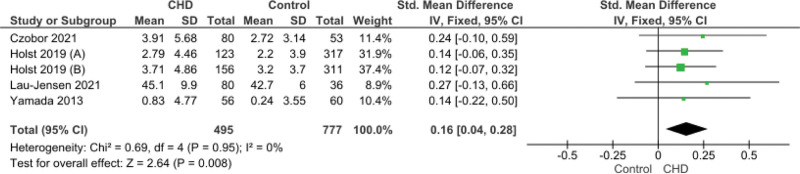
Forest plot illustrating pooled ADHD Hyperactivity-Impulsivity Subscale (informant). ADHD = attention-deficit/hyperactivity disorder.

This measures the difficulty impulsive behaviors that result in interfering with completing tasks, interrupting people, forgetting things a child asking to do, etc. This score reported in 4 studies with a significant increase of the score in CHD group (SMD = 0.16, 95% CI [0.04, 0.28], *P* = .008). The pooled data were homogeneous (*P* = .95, I^2^ = 0%) (Fig. 3).

### 3.5. ADHD Inattention Subscale (informant)

**Figure 4.**
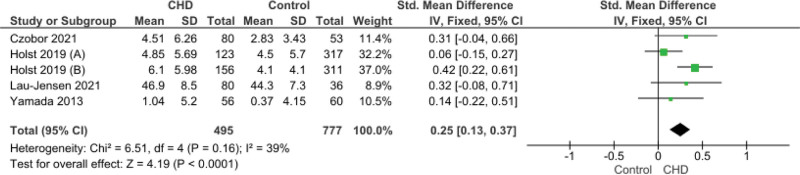
Forest plot illustrating pooled ADHD Inattention Subscale (informant). ADHD = attention-deficit/hyperactivity disorder.

This score reported in 4 studies. The pooled data showed that significant increase of this score in CHD group (SMD = 0.25, 95% CI [0.13, 0.37], *P* < .001). Pooled data were homogeneous (*P* = .16, I^2^ = 39%) (Fig. 4).

### 3.6. ADHD Combined Score (informant)

**Figure 5.**
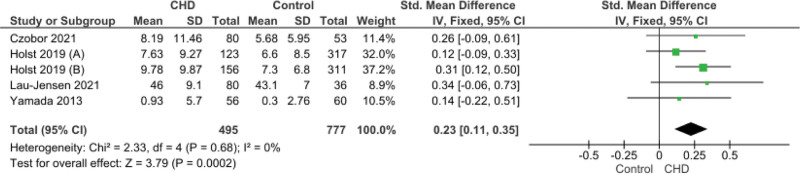
Forest plot illustrating pooled ADHD Combined Score (informant). ADHD = attention-deficit/hyperactivity disorder.

ADHD Combined Score (informant) have been used in patients meeting the criteria of both ADHD inattentive type and hyperactive/impulsive type. This score reported in 4 studies. The pooled data showed that significant increase of this score in CHD group (SMD = 0.23, 95% CI [0.11, 0.35], *P* = .0002). Pooled data were homogeneous (*P* = .68, I^2^ = 0%) (Fig. 5).

## 4. Discussion

To our knowledge, this is the first meta-analysis to estimate the severity of ADHD symptoms in congenital heart disease patients; Our results showed significant increases in the severity of ADHD symptoms in CHD patients more than controls with a strong association between them in general (ADHD—combined, ADHD—inattention, and ADHD—hyperactivity-Impulsivity) which stresses the importance of early detection of ADHD in children with CHD in the clinical practice and periodic reevaluation every couple of years or following any new cardiac event or surgery.

Moreover, most of the available data reported the prevalence of ADHD in different subtypes of CHD but failed to focus on the severity of ADHD. All factors that can affect the brain development will play a role in the development of neuropsychological deficits (e.g., Prenatal factors such as alcohol, nicotine, and other substances or children born prematurely or injuries, traumas, etc) CHDs may result in reduced cerebral blood flow and oxygen to the brain, which can affect how the brain develops prenatally and in early childhood^[15,16]^ regarding the factors affect the severity of ADHD symptoms in patients with CHDs, our findings showed that the symptoms worsen with age and complications of heart surgeries, placement of ventricular assist devices, and transplant. The age at the time of cardiac surgery makes a difference too, the lower the age at operation, the better the outcomes.^[17,18]^

According to the American Psychiatric Association, ADHD symptoms come with 3 presentations: ADHD—combined, ADHD—inattention, and ADHD—hyperactivity. Many different types of scales are available to determine the type and assess the severity of ADHD. Our studies showed a significant increase in ADHD index T score, ADHD Hyperactivity-Impulsivity Subscale, ADHD Inattention Subscale (informant), and ADHD Combined Score (informant) in CHD patients more than the controls. DeMaso et al^[19]^ compare adolescents with d-transposition of the great arteries (d-TGA) with healthy adolescents, the results showed 19% of adolescents with repaired d-TGA met Diagnostic and Statistical Manual of Mental Disorders, Fourth Edition (DSM-IV) criteria for ADHD with a significantly higher mean of T score in the d-TGA group than in the referent group (*P* = .01), More recently, DeMaso et al^[20]^ examine psychiatric disorders in adolescents with single ventricle CHD and whether the patient-related risk factors contribute to that dysfunction. They reported a stunning 55% met the DSM-IV lifetime criteria for anxiety disorder and 53% met the criteria for ADHD among adolescents with single-ventricle CHD and their results end up with Adolescents with single ventricle CHD have a high risk of psychiatric morbidity specifically with anxiety disorder and ADHD (*P* < .001 each) and early identification of psychiatric symptoms is critical for better management. Regarding patient-related risk factors, they find that open-cardiac surgeries or age at the first cardiac surgery, are important for better psychosocial outcomes.

In 2017, Holland et al^[21]^ assess psychiatric disorders in adolescents with repaired tetralogy of Fallot (TOF) and end up with Adolescents with TOF either without or with an origin of a genetic defect had a higher lifetime prevalence of ADHD than referents (19% and 39%, respectively, vs 5%; *P* = .04 and.002, respectively). They also examined associations between psychiatric status and physical illness severity; the median total severity scores on the BPRS-C (The Brief Psychiatric Rating Scale for Children) were low for all group but were significantly higher in adolescents with TOF and a genetic diagnosis. With respect to ADHD symptoms, adolescents with TOF without or with a genetic diagnosis scored higher than referents on CADS ADHD Index (*P* < .0001 for both). Lau-Jensen et al^[22]^ investigate the distribution in ADHD symptoms in young adults with simple CHD. They reported 15% of all T-scores were above 65 In the CHD group to 4% of the T-scores in the control group and end up with patients with a simple CHDs have a higher symptom burden across all ADHD scores (ADHD—combined: *P* = .007, ADHD—inattention: *P* = .002, and ADHD—hyperactivity: *P* = .03) and all symptom sub-scores (inattention/memory problems: *P* = .001, hyperactivity/restlessness: *P* = .03, impulsivity/emotional lability: *P* = .001) therefore they emphasize the importance of the routine screening for ADHD symptoms to facilitate adequate help as these symptoms are easily overlooked. Czobor et al^[23]^ investigate the differences in ADHD symptoms between children who underwent cardiac surgery at different ages and controls. The results indicted better outcomes with the lower age at the first cardiac surgery and significantly higher severity of ADHD symptoms in children who underwent cardiac surgery at or above the age of 3 compared to non-operated children or children who operated at a younger age.

Holst et al^[24]^ investigate if children with CHD have an increased prevalence of ADHD symptoms compared with the healthy controls and their results like previous studies in increasing the ADHD frequency among children with CHDs who had significantly increased inattention scores (*P* = .009) and total ADHD scores (*P* = .008) compared with controls. Yamada et al aim to determine if school-aged children underwent early heart surgery for CHD are more likely than healthy controls to have screening scores on the Swanson, Nolan, and Pelham IV (SNAP-IV) questionnaire suggestive of ADHD, and the results supported the significant potential increased risk of ADHD in children undergoing early surgery of CHD. Our study should be interpreted considering some limitations. First, our data is derived from observational data and retrospective studies. Second, the baseline characteristics between the groups were not equivalent with small number of CHD patients and larger number of healthy individuals; however, we observed no heterogeneity in most of our analyses and if heterogeneity was observed it was solved. Lastly, the outcome scores reported are based on informant data which make them vulnerable to subjective changes from practitioner and the other and from center and the other.

Moreover, several factors have been connected to the development of ADHD in patients with CHD. First is the genetic predisposition. A study conducted in 2010 found that multiple genes have been associated with an increased risk for ADHD, and some of these genes may also play a role in heart development,^[25]^ while another found that there is a tendency for certain conditions to run in families. Families with a history of ADHD or CHD may be at an increased risk of having children with these conditions^[26]^; moreover, a meta-analysis in 2018 found that the health of the mother during pregnancy can impact the risk of both ADHD and CHD with maternal health conditions and genetic factors may contribute to both conditions.^[27]^

## 5. Conclusion

Our study revealed a strong association between CHD and not only ADHD, but also the severity of ADHD making early diagnosis of ADHD in children with CHD a mandatory step in the clinical evaluation practice to improve these children on both clinical and psychological aspects.

## Author contributions

**Conceptualization:** Mohammed Tarek Hasan.

**Data curation:** Mohammed Tarek Hasan.

**Formal analysis:** Mohammed Tarek Hasan, Mahmoud Shaban Abdelgalil, Ali Elkhadragy.

**Investigation:** Mohammed Tarek Hasan, Mahmoud Shaban Abdelgalil, Merihan A. Elbadawy, Amr Mahmoud Elrosasy, Ali Elkhadragy, Mahmoud El Garhy.

**Methodology:** Mohammed Tarek Hasan, Mahmoud Shaban Abdelgalil, Merihan A. Elbadawy, Amr Mahmoud Elrosasy, Mahmoud El Garhy.

**Project administration:** Mohammed Tarek Hasan

**Software:** Mohammed Tarek Hasan.

**Supervision:** Ahmed K. Awad.

**Validation:** Mahmoud El Garhy.

**Visualization:** Mohammed Tarek Hasan.

**Writing – original draft:** Mohammed Tarek Hasan, Mahmoud Shaban Abdelgalil, Merihan A. Elbadawy.

**Writing – review & editing:** Mohammed Tarek Hasan.
